# Comprehensive analysis of the immune pattern of T cell subsets in chronic myeloid leukemia before and after TKI treatment

**DOI:** 10.3389/fimmu.2023.1078118

**Published:** 2023-01-19

**Authors:** Danlin Yao, Jing Lai, Yuhong Lu, Jun Zhong, Xianfeng Zha, Xin Huang, Lian Liu, Xiangbo Zeng, Shaohua Chen, Jianyu Weng, Xin Du, Yangqiu Li, Ling Xu

**Affiliations:** ^1^ Key Laboratory for Regenerative Medicine of Ministry of Education, Institute of Hematology, School of Medicine, Jinan University, Guangzhou, China; ^2^ Department of Hematology, The Second Affiliated Hospital, Guangzhou Medical University, Guangzhou, China; ^3^ Department of Hematology, First Affiliated Hospital, Jinan University, Guangzhou, China; ^4^ Department of Clinical Laboratory, First Affiliated Hospital, Jinan University, Guangzhou, China; ^5^ Department of Hematology, Guangdong Provincial People’s Hospital, Guangdong Academy of Medical Sciences, Guangzhou, China

**Keywords:** T cell subsets, CML, bone marrow microenvironment, immunological phenotypes, tyrosine kinase inhibitor

## Abstract

**Background:**

Immunological phenotypes and differentiation statuses commonly decide the T cell function and anti-tumor ability. However, little is known about these alterations in CML patients.

**Method:**

Here, we investigated the immunologic phenotypes (CD38/CD69/HLA-DR/CD28/CD57/BTLA/TIGIT/PD-1) of T subsets (TN, TCM, TEM, and TEMRA) in peripheral blood (PB) and bone marrow (BM) from de novo CML patients (DN-CML), patients who achieved a molecular response (MR) and those who failed to achieve an MR (TKI-F) after tyrosine kinase inhibitor (TKI) treatment using multicolor flow cytometry.

**Results:**

CD38 or HLA-DR positive PB CD8+TN and TCM cells decreased in the DN-CML patients and this was further decreased in TKI-F patients. Meanwhile, the level of PD-1 elevated in CD8+ TEM and TEMRA cells from PB in all groups. Among BM sample, the level of HLA-DR+CD8+TCM cells significantly decreased in all groups and CD8+TEMRA cells from TKI-F patients exhibited increased level of TIGIT and CD8+ tissue-residual T cells (TRM) from DN-CML patients expressed a higher level of PD-1 and TIGIT. Lastly, we found a significantly decreased proportion of CD86+ dendritic cells (DCs) and an imbalanced CD80/CD86 in the PB and BM of DN-CML patients, which may impair the activation of T cells.

**Conclusion:**

In summary, early differentiated TN and TCM cells from CML patients may remain in an inadequate activation state, particularly for TKI-F patients. And effector T cells (TEM, TEMRA and TRM) may be dysfunctional due to the expression of PD-1 and TIGIT in CML patients. Meanwhile, DCs cells exhibited the impairment of costimulatory molecule expression in DN-CML patients. Those factors may jointly contribute to the immune escape in CML patients.

## Introduction

Chronic myeloid leukemia (CML) is a hematological tumor driven by the BCR-ABL1 fusion protein, which constitutively activates tyrosine kinases. This activation leads to the accumulation of immature granulocytes and their progenitors in peripheral blood (PB) and bone marrow (BM). The advent of tyrosine kinase inhibitors (TKIs) has transformed CML into a chronic disease. Most patients achieve a life expectancy close to that of the general population (1–5). However, some patients do not have an optimal response in the initial stage of TKI treatment or lose a previously achieved hematological, cytogenetic, or molecular response during TKI treatment. These patients are identified as failing response to TKIs (TKI-F) and frequently develop into the accelerated phase (AP) or blast crisis (BC) phase, leading to poor prognosis (6–8). Additionally, due to the side effects and costs of TKIs, many CML patients who achieve major molecular remission (MMR) are eager to withdraw from the drug. Currently, studying the immune system changes in those patients before and after TKI treatment may provide more information for solving their problems.

T cells play an integral role against pathogens and clear tumor cells. During the immune process, naïve T (T_N_) cells recognize pathogens presented by dendritic cells (DCs) and accept activation signals by binding the costimulatory ligand CD80 or CD86 on DCs. T_N_ cells further differentiate into effector memory T cells (T_EM_)/CD45RA^+^ effector memory T cells (T_EMRA_) to clear antigens. Once pathogens were cleared, most activated T cells experienced apoptosis and a minority of survival effector T cells becomes central memory T cells (T_CM_) cells. Afterward, T_CM_ cells provide immediate protection when re-infected and ultimately persist for a lifetime (9–13). Thus, the different differentiated status of the T cell subsets partially indicates the function of T cells. Recently, increasing evidence has indicated that the immunological status of T cell subsets provides better prognostication than CD4^+^ or CD8^+^ T cells in cancer patients, e.g., AML patients who express a higher percentage of PD-1^+^Tim3^+^CD8^+^ T_CM_ cells are prone to relapse, and the prognosis of breast cancer patient with a higher number of CD8^+^ tissue residual memory T (T_RM_) cells was better (14, 15).

Indeed, accumulating evidence has proven that CML patients undergo several phenotypic and functional aberrations in the immune system, and this phenomenon is applicable to CML patients who at diagnosis, achieve MMR on TKI therapy and even at treatment-free remission (16–20). It’s well known that the activation and proliferation of T cells are impaired due to the lower expression of CD3ζ and higher expression of immune checkpoints (ICs) (18, 21, 22). However, most studies only focused on total CD8^+^ or CD4^+^ T cells, and exploration of the immunophenotypes of T cell subsets remains limited for CML patients, particularly TKI-F patients. Additionally, BM is the origin and natural shelter for leukemia cells. Moreover, the BM accumulates immunosuppressive cells, including regulatory T (Treg) cells, myeloid-derived suppressor cells (MDSCs), and plasmacytoid DC that inhibit the anti-tumor response of T cells (23–27). These characteristics make the BM microenvironment (BMM) similar to the immunologic microenvironment of solid tumors. Hence, intensive study of the immunophenotypic characteristics of the T cell subsets driven by the leukemia BMM is critical for providing effective immunotherapy for CML patients.

Here, we used flow cytometry to assay the expression of the activation markers CD38, CD69, and human leukocyte antigen – DR isotype (HLA-DR), the IC molecules programmed death-1 (PD-1), B, and T lymphocyte attenuator (BTLA), and T cell immunoglobulin and ITIM domain (TIGIT), the co-stimulation marker CD28, and the immune senescence marker CD57 on different T cell subsets in PB and BM from CML patients. We categorized CML patients into different statuses according to the level of BCR-ABL1 and TKI-treatment response: *de novo* CML (DN-CML: BCR-ABL1 > 10%), molecular remission (MR: BCR-ABL1 < 10%), and TKI-F. The TKI-F patients were identified as CML patients who failed to achieve a molecular response (TKI-F, BCR-ABL1 > 10%) with regular oral administration of first or second-generation TKIs after 3 months. Finally, we describe the T cell costimulatory molecules CD80 and CD86 on DCs in the CML groups.

## Materials and methods

### Patient samples

PB samples were obtained from DN-CML (n = 16), TKI-F (n = 9), and MR (n = 20) patients. BM aspirate samples were extracted from 23 cases, including 11 newly diagnosed patients, 6 at MR, and 6 at TKI-F. PB samples were obtained from healthy individuals (HIs; n =12), BM aspirate samples from hematopoietic stem cell transplantation (HSCT) donors (n = 6) and patients with iron-deficiency anemia (n = 3) were collected as control. All MR patients achieved complete hematologic response (CHR) with BCR/ABL < 10% after TKI treatment. In addition, previous studies have found that the immunologic characteristics of T cells in CML patients varied with different molecular remission levels (18). The MR patients were further divided into 2 groups according to the BCR/ABL1 level. MMR patients (n = 10) with a level ≤ 0.1% and pre-MMR (n = 10) representing the period before MMR was achieved with a BCR-ABL1 transcript level > 0.1% and < 10%. The TKI-F were patients with BCR/ABL1 > 10% consistently after regular 12-month TKI treatment. Sample data are shown in **Tables 1**, **2**. Detailed sample information of TKI-F patients are shown in **Table 3**.

**Table 1:** Peripheral blood sample characteristics.

		DN-CML	TKI-F	Pre-MMR	MMR	HI
Cases		16	9	10	10	12
Status		CP/BC(14/2)	CP/AP/BC (4/3/2)	CHR	CHR	_
Age (median; range)		45.5 (32-74)	48 (35-68)	39.5 (28-79)	40.5 (21-79)	42.5 (21-74)
Gender (male/female)		9/7	7/2	6/3	3/6	6/6
Diagnosis data (median, range)						
BCR-ABL1 (IS)%		95.6 (13.4-240.0)	32 (11.8-194.4)	2.35 (0.005-9.1)	0.029 (0.028-0.09)	
TKI duration (median, range) months		_	28 (14-120)	7 (1-83)	47 (5-108)	

DN, de novo; CP, chronic phase; AP, acceleration phase; BC, blast crisis; CHR, clinical hematologic remission; IS, international standard.

**Table 2:** Bone marrow sample characteristics.

	DN-CML	TKI-F	MR	HI
Cases	11	6	6	9
Status	CP/AP (10/1)	CP/AP/CHR (1/2/3)	CHR	CHR
Age (median; range)	45 (32-74)	42.5 (35-68)	43 (25-79)	35.5 (19-62)
Gender (male/female)	7/4	4/2	3/3	4/5

DN, de novo; CP, chronic phase; AP, acceleration phase; BC, blast crisis; CHR, clinical hematologic remission; IS, international standard.

**Table 3:** TKI-F PB sample characteristics.

	Age/ Gender	Status	BCR-ABL 1 (IS) %	Mutation in ABL1 kinase region	TKI- Duration(months)	TKI- drug
P1	46/M	BC	89.723	N	14	Imatinib
P2	50/M	AP	124.154	T315I	50	Nilotinib
P3	48/M	AP	118.073	N	26	Dasatinib
P4	35/M	BC	45.931	N	20	Dasatinib
P5	51/M	AP	49.931	F317I	96	Imatinib
P6	56/F	CP	13.732	c.1423_1424ins35 (p.Cys475fs*11)	28	Nilotinib
P7	35/M	CP	194.110	N	17	Imatinib
P8	39/F	CP	58.121	T315I	40	Imatinib
P9	68/M	CP	11.843	N	120	Imatinib

CP, chronic phase; AP, acceleration phase; BC, blast crisis; IS, international standard.

### Flow cytometry analysis

PB and BM samples were collected in EDTA tubes. First, 150 µl of PB or BM aspirate was incubated with CCR7-BV421 for 15 min in the dark at 37°C. Then, the samples were incubated with multiple premixed fluorescence antibodies for 20 min in the dark at room temperature. The final volume was 200 µl. T cells subsets and surface antibodies staining were performed in two 11-color panels including the following antibodies. CD45-BUV395 (clone HI30, BD) was used to identify CD45^high^ cells which can rule out tumor cells. CD3-AF700 (clone UCHT1, BD), CD4-APC-H7 (clone RPA-T4, BD), CD8-APC-H7 (clone SK1, BD), CD45RA-Percp-cy5 (clone HI100, Biolegend), CCR7-BV605 (clone 3D12, BD) and CD69-PE-cy7 (clone FN50, BD) were used to identify CD4^+^ or CD8^+^ T subsets. CD38-APC (clone HIT2, BD), BTLA-PE-CF594 (clone J168-540, BD), TIGIT-BV421 (clone A15153G, Biolegend) and CD28-BB515 (clone CD28.2, BD) were used in Tube 1, CD57-APC (clone NK-1, BD), PD-1-BV421 (clone EH12.2H7, Biolegend), and HLA-DR-PE-CF594 (clone G46-6, BD) were used in Tube 2. DCs cells and surface antibodies staining were performed in 5-color panels including the following antibodies. CD45-BUV395 (clone HI30, BD), HLA-DR-PE-CF594 (clone G46-6, BD), Lin-FITC (CD3, CD14, CD16, CD20, CD56, cat:340546, BD), CD80-PE (clone L307.4, BD), CD86-PE-cy7 (clone FUN-1, BD). The samples were lysed using lysis buffer (BD; Cat: 555899) for 15-20 min and washed and suspended in phosphate buffer saline (PBS). Finally, 20 µl of absolute count microsphere (Thermos; Cat: C36950) were added to the samples to calculate the absolute number of cells. A minimum of 20,000 CD3^+^ T cells and 2000 DC cells were acquired by flow cytometry (FACS Fortessa, BD Bioscience) and analyzed using Flowjo 10.6. FCS.

### Statistical analysis

All data were represented as medians, and differences between every two groups were analyzed by the Mann-Whitney U test. The statistical analysis and figure generation were performed using GraphPad Prism version 8.02 software. Significance is indicated as *P* < 0.05.

## Results

### A higher percentage of PD-1^+^CD8^+^ T cells in the PB of CML patients of different statuses

**Figure 1:**
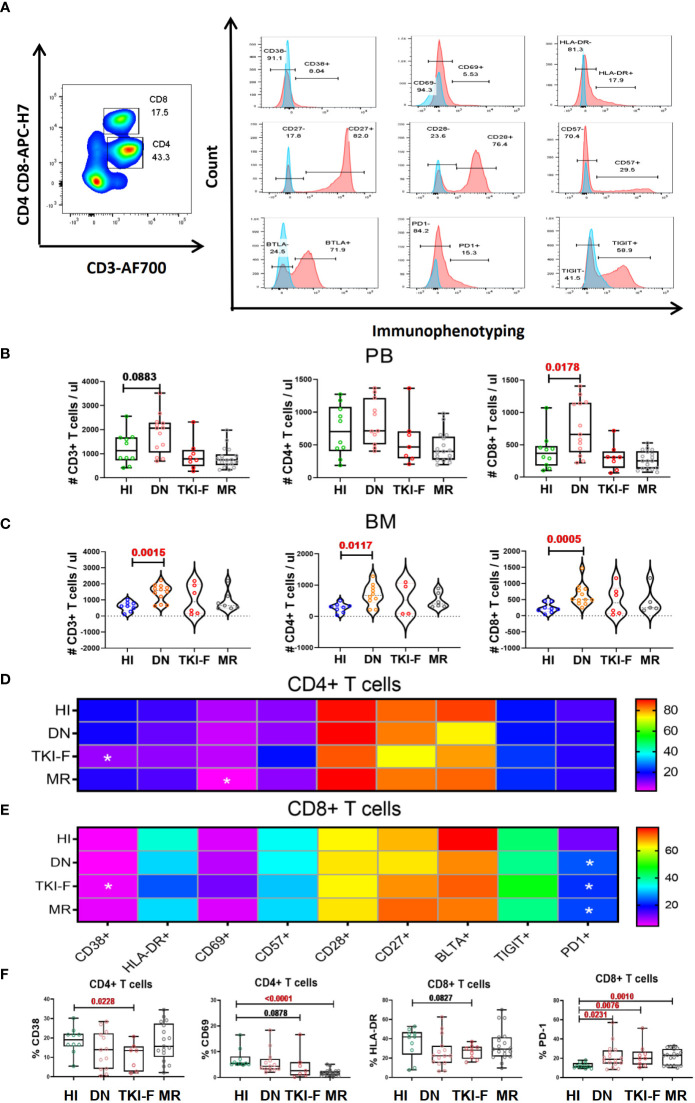
TKI-F patients exhibit a lower level of the activation markers CD38 and CD69 on CD4^+^ T cells and HLA-DR on CD8^+^ T cells, and the exhaustion marker PD-1 increased on PB-CD8^+^ T cells in all CML groups. **(A)** The top figure shows the gating strategy for CD38, CD69, HLA-DR, CD28, CD57 BTLA, TIGIT, and PD-1 in the CD4^+^ and CD8^+^ populations by flow cytometry. The absolute number of CD3^+^, CD4^+^, and CD8^+^ T cells in the PB and BM **(B, C)** The darkness of the color represents the mean frequency of a single immune marker on CD4^+^ **(D)** and CD8^+^ **(E)** T cells in PB from HIs and DN-CML, TKI-F, and MR patients. The asterisk (*) represents a significant alteration in CML patients compared with HIs. **(F)** The proportion of CD38^+^CD4^+^, CD69^+^CD4^+^, HLA-DR^+^CD8^+^, and PD-1^+^CD8^+^ in PB from HIs and DN-CML, TKI-F, and MR patients. HIs-PB (CD4, n = 12, CD8, n = 12), DN-CML-PB (CD4, n = 12, CD8, n = 16), TKI-F-PB (CD4, n = 7, CD8, n = 9), and MR-PB (CD4, n = 17, CD8, n = 20). The *P* values shown are from the Mann-Whitney U test between groups.

The gating strategy for identifying the CD4+ and CD8+ T cells and their phenotypic characteristics was shown in **Figure 1A**. We first identified the absolute number of CD3^+^, CD4^+^, and CD8^+^ T cells in PB and BM in each CML group and found that there was a slightly increased trend for CD3^+^ T cells (1,133 cells/μl vs 2,064 cells/μl, *P* = 0.0883) and a significant increase in CD8^+^ T cells in the PB of DN-CML patients compared with HIs (368 cells/μl vs 1,581 cells/μl, *P* = 0.0178) (**Figure 1B**). In the BM, CD3^+^ (616 cells/μl vs 1320 cells/μl *P* = 0.0015), CD4^+^ (301 cells/μl vs 671 cells/μl, *P* = 0.0117), and CD8^+^ (210 cells/μl vs 507 cells/μl, *P* = 0.0005) T cells were all significantly increased in DN-CML patients compared with HIs (**Figure 1C**). No significant differences were observed between other CML groups and HIs.

Next, we compared the expression of CD38, CD69, HLA-DR, CD28, CD57, BTLA, TIGIT, and PD-1 on the CD4^+^ and CD8^+^ T subsets in PB for each CML group. The results demonstrated that the expression of the activation marker CD38 on the CD4^+^ T subset was significantly decreased in TKI-F patients compared with HIs (19.6% vs 10.5%, *P* = 0.0020) (**Figures 1D and F**), while the level of CD69 and HLA-DR also showed a decreasing trend on the CD4^+^ and CD8^+^ T subset respectively (**Figure 1F**). These alterations suggest that the activation capacity of CD4^+^ and CD8^+^ T cells from TKI-F patients may be impaired. In addition, a lower level of CD69^+^CD4^+^ T cells (5.46% vs 1.89%, *P* < 0.0001) was found in MR patients compared to HIs (**Figures 1D, F**). For the exhausted and senescent molecular expression pattern, we found that the level of PD-1^+^CD8^+^ T cells was significantly increased in DN-CML (25.3% vs 16.5%, *P* = 0.0231), TKI-F (24.6% vs 16.5%, *P* = 0.0076), and MR (23.8% vs 16.5%, *P* = 0.0016) patients when compared with HIs (**Figure 1F**).

### An increased PD-1 level in the CD8^+^ T_EM_ and T_EMRA_ subsets in PB from CML patients

**Figure 2:**
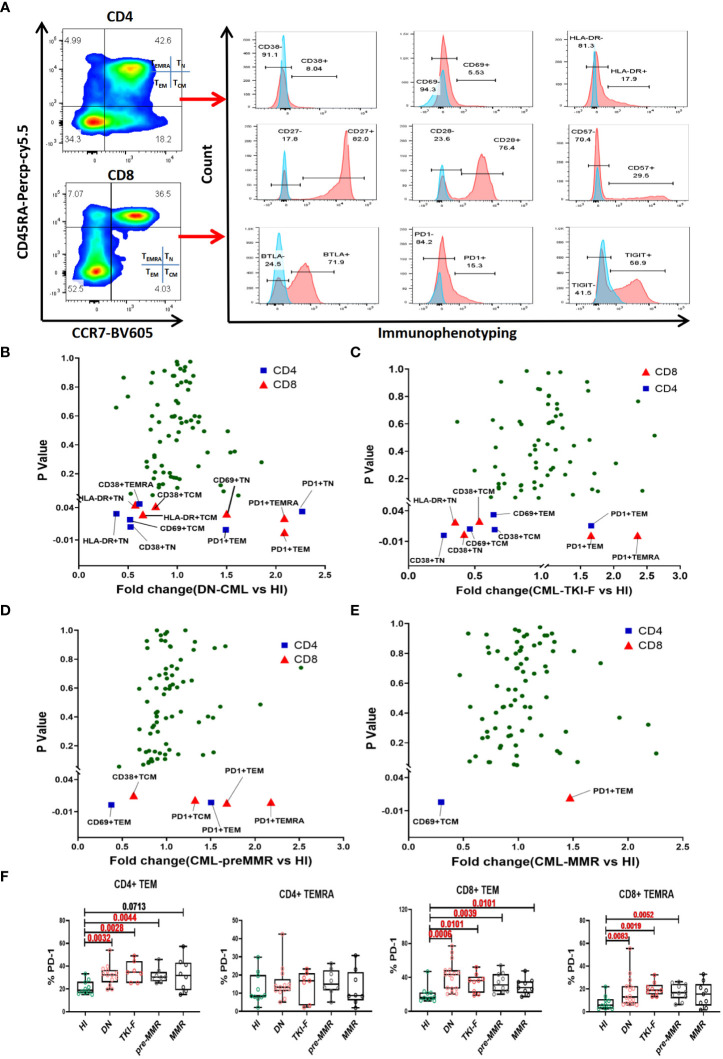
The abnormal immunophenotype of PB T cell subsets distributed in DN-CML and TKI-F patients gradually returning to normal in pre-MMR and MMR groups. **(A)** Gating strategy for the CD4^+^ and CD8^+^ T cells subsets in one HI. CD45RA and CCR7 were used to divide the T cells into T_N_ (CD45RA^+^CCR7^+^), T_CM_ (CD45RA^-^CCR7^+^), T_EM_ (CD45RA^-^CCR7^-^), and T_EMRA_ (CD45RA^+^CCR7^-^) cells. The proportion of the T cell subset single-marker immunophenotypes in HIs and DN-CML **(B)**, TKI-F **(C)**, pre-MMR **(D)**, and MMR **(E)** patients were compared (Mann-Whitney U test). The *P-value* plotted on the vertical axis of volcano figure. Immunologic characteristics with the mean percentage of fold change (FC) > 1 are enriched in CML patients, and FC < 1 was more frequent in HIs. The blue and red points respectively represent CD4^+^ and CD8^+^ T cells. **(F)** The frequency of PD-1 on CD4^+^ T_EM_, CD4^+^ T_EMRA_, CD8^+^ T_EM,_ and CD8^+^ T_EMRA_ cells in PB from HIs and DN-CML, TKI-F, pre-MMR, and MMR patients. HIs-PB (CD4, n = 10, CD8, n = 10), DN-CML-PB (CD4, n = 13, CD8, n = 16), TKI-F-PB (CD4, n = 7, CD8, n = 9) and MR-PB (CD4, n = 18, CD8, n = 20). The *P* values shown are from the Mann-Whitney U test between groups.

To further understand the immunophenotypic alterations in each T cell subset. We divided the CD4^+^ and CD8^+^ T cells into T_N_ (CD45RA^+^CCR7^+^), T_CM_ (CD45RA^-^CCR7^+^), T_EM_ (CD45RA^+^CCR7^-^), and T_EMRA_ (CD45RA^-^CCR7^-^) subsets based on CD45RA and CCR7 expression. We compared activated/inhibitory/senescent phenotypic characteristics of each subset in the PB of the patient groups and HIs. The gating strategy is shown in **Figure 2A**. To exhibit the differences of a single marker in the T cell subsets, the fold change (FC) of the mean value between each CML group and HIs was shown in volcano figures (**Figures 2B-E**). We found that the expression of the activation markers CD38, CD69, and HLA-DR decreased on T_N_ and T_CM_ subsets in DN-CML patients and further decreased in TKI-F patients. Moreover, these abnormalities gradually restored to normal levels at the time of remission. The detailed expression characteristics of each group were shown in **Supplementary Figure 1**. Unlike T_N_ and T_CM_, which exhibit a lower level of activation markers, the T_EM_ and T_EMRA_ subsets mainly demonstrate increased expression of PD-1 in the PB of the CML patient groups. (**Figures 2B-E**). We further compared PD-1 expression on T_EM_ and T_EMRA_ cells between the CML patients and HIs. The percentage of PD-1^+^CD4^+^ T_EM_ cells significantly increased in DN-CML (31.54%, *P* = 0.0032), TKI-F (35.47%, *P* = 0.0026), and Pre-MMR (31.7%, *P* = 0.0044) patients compared to HIs (21.2%). No significant difference was observed in the CD4^+^ T_EMRA_ subset between CML patients and HIs. Similarly, the proportion of PD1^+^CD8^+^ T_EM_ cells increased in DN-CML (41.90%, *P* = 0.0006), TKI-F (33.28%, *P* = 0.0101), Pre-MMR (33.75%, *P* = 0.0039), and even MMR (29.56%, *P* = 0.0101) patients compared with HIs (20.08%). In addition, the level of PD-1^+^CD8^+^ T_EMRA_ significantly increased in DN-CML (16.57%, *P* = 0.0083), TKI-F (18.73%, *P* = 0.0019), and Pre-MMR (17.32%, *P* = 0.0052) patients but returned to a normal level in some MMR patients (15.27%, *P* = 0.3686) when compared with HIs (7.94%) (**Figure 2F**).

### Increased PD-1^+^/TIGIT^+^CD8^+^ T_RM_ cells in BM of DN-CML patients

The immunosuppressive BMM protects malignant hematopoietic stem cells from immunological surveillance, which may contribute to leukemia relapse (23). We examined the expression of each marker on BM CD8^+^ T cells and subsets. The results revealed no significant difference in the expression of each of the above markers on total CD8^+^ T cells between each CML group and HIs. However, when looking at the subset level, we found a significantly decreased level of HLA-DR^+^CD8^+^T_CM_ in DN-CML (36.75% vs 12.40%, *P* = 0.0462) and a further decrease in TKI-F (36.75% vs 6.71%, *P* = 0.0087) and MR (37.75% vs 5.20%, *P* = 0.0082) patients compared to the control group. For other markers, only an increased percentage of TIGIT^+^CD8^+^ T_EMRA_ (42.60% vs 75.45%, *P* = 0.0256) was observed in TKI-F patients (**Supplementary Figure 2**).

**Figure 3:**
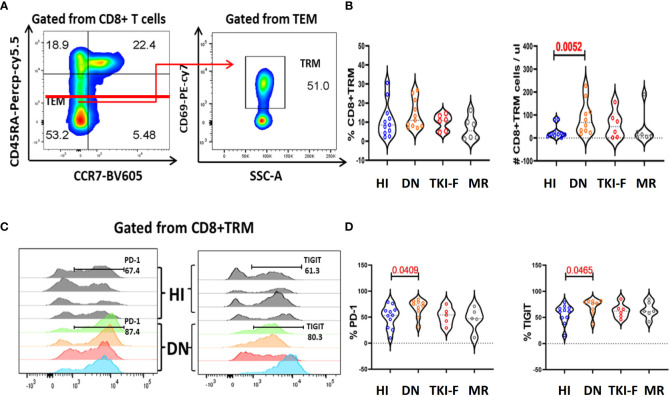
The absolute numbers of CD8^+^T_RM_ cells increased in the BM of DN-CML patients while accompanied by an elevated expression of PD-1 and TIGIT. **(A)** Gating strategies to indicate the T_RM_ cells. CD45RA^-^ and CCR7^-^ were used to identify the T_EM_ subsets and then T_RM_ cells were further gated by CD69 expression. **(B)** The percentage and absolute number of CD8^+^T_RM_ cells in HIs, DN-CML, TKI-F, and MR patients. **(C)** Flow-cytometry analysis detected the frequency of PD-1^+^CD8^+^ T_RM_ (left) and TIGIT^+^CD8^+^ T_RM_ (right) in HIs (top: n = 4) and DN-CML (below: n = 4). **(D)** Increased proportion of PD-1 (left) and TIGIT (right) on CD8^+^ T_RM_ cells in BM from HIs (n = 10) and DN-CML (n = 10), TKI-F (n = 6), and MR (n = 6) patients. The *P* values shown are from the Mann-Whitney U test between groups.

With the exception of the classic memory T cell subsets, we also examined the expression of the above markers on T_RM_ cells, which are abundant in non-lymphoid tissues, such as skin, lung, and BM (28). T_RM_ cells express a low level of CD45RA and lack CCR7, and CD69 is a key marker to identify T_RM_ (CD45RA^+^CCR7^-^CD69^+^) from T_EM_ cells (29). Detailed gating strategies are shown in **Figure 3A** and the expression of PD-1 and TIGIT in the BM of HI and CML patients were shown in **Figure 3C**. These results demonstrated that the number of CD8^+^ T_RM_ cells increased in DN-CML patients but there was no difference in the TKI-F and MR groups compared to HIs (**Figure 3B**). Further, we also found a significantly increased percentage of PD-1^+^CD8^+^ T_RM_ cells (53.50% vs 73.30%, *P* = 0.0409) and TIGIT^+^CD8^+^ T_RM_ (61.40% vs 77.00%, *P* = 0.0465) cells in DN-CML patients compared with HIs (**Figure 3D**).

### Decreased expression of CD86 on DC cells in PB and BM from DN-CML patients

**Figure 4:**
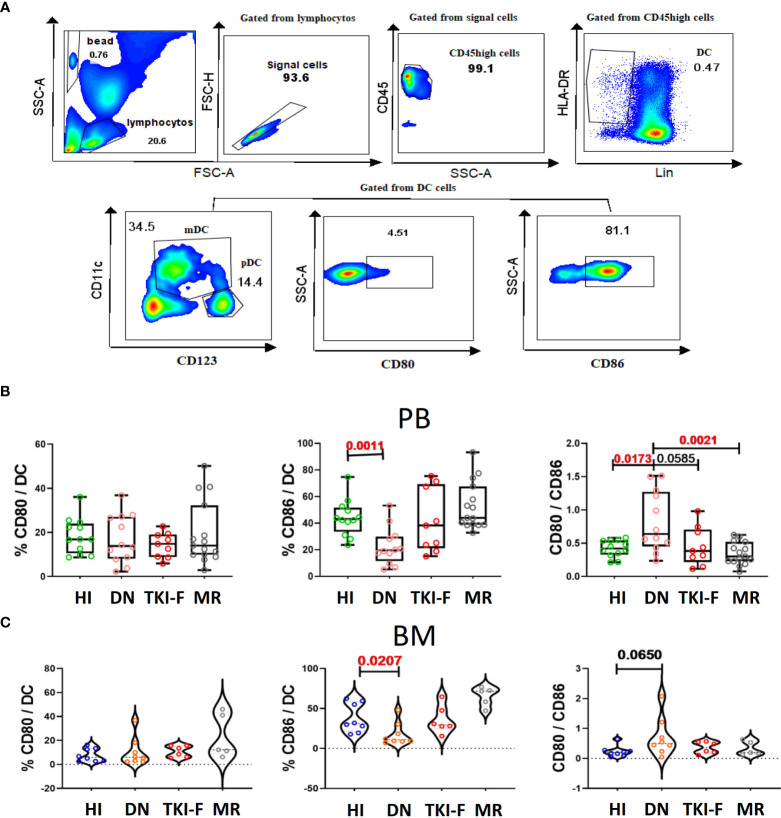
PB and BM DCs cells from DN-CML patients express a lower percentage of CD86 and with an unbalance CD80/CD86 ratio. **(A)** Gating strategy to identify DCs cells and the expression of CD80/CD86 on DCs cells. The CD45^high^ population was used to eliminate immature cells, and then HLA-DR^+^ and Lin^-^ (CD3, CD19, CD56, CD14 and CD16) was used to identify that DCs. CD80^+^ and CD86^+^ were further used to gate CD80^+^ DCs and CD86^+^ DCs cells. **(B)** The frequency of CD80^+^ DC, CD86^+^ DC, and CD80/CD86 ratio in PB **(B)** and BM **(C)** of HIs (PB, n = 12, BM, n = 8) and DN-CML (PB, n = 12, BM, n = 8), TKI-F (PB, n = 9, BM, n = 6), and MR (PB, n = 17, BM, n = 5) patients. The *P* values shown are from the Mann-Whitney U test between groups.

DCs can provide costimulatory signals driven by the molecules CD80 and CD86 to induce T cell activation and functional differentiation. Here, we identified DCs (HLA-DR^+^Lin^-^) from the CD45^high^ population aiming to eliminate the interference from leukemia cells. Next, we analyzed the expression of CD80 and CD86 on DCs, and detailed gating strategies are presented in **Figure 4A**. The results show that the percentage of CD86^+^ DCs decreased in PB (41.2% vs 21.5%, *P* = 0.0011) and BM (30.9% vs 12.45%, *P* = 0.0207) of DN-CML patients compared to controls. Previous studies have reported that DCs expressing a normal level of CD80 and lower CD86 act as immature DCs. We further assayed CD80 and CD86 on T cells in the CML groups and HIs. The result demonstrated a significantly decreased ratio of CD80/CD86 both in the PB (0.42 vs 0.90, *P =* 0.0173) and BM (0.19 vs 0.36, *P* = 0.0650) of DN-CML patients, and 5 patients had an inverse ratio. Additionally, the CD86 expression and CD80/CD86 ratio could return to a normal level after TKI treatment (**Figures 4B, C**).

## Discussion

Our previous study found that memory T cell subset distribution skewed toward a terminally differentiated status in DN-CML patients and restore in MR-CML patients, suggesting that the T cell subset distribution might be important for inducing and maintaining remission in CML patients (30). In this study, we further found that the immunophenotype of the T cell subsets (T_N_, T_CM_, T_EM_, T_EMRA_, T_RM_) was associated with the disease status and location. On the level of the total CD4^+^ and CD8^+^ population, we only found a few function markers or even no markers were changed in the PB and BM of patients respectively, however, further analysis of the T cell subsets revealed that the markers representing the activation and proliferation (CD38, HLA-DR, and CD69) (31, 32) were decreased in the less differentiated T_N_ and T_CM_ subsets in the DN-CML and TKI-F patients, while gradually recovered in the pre-MMR and MMR patients. In addition, the higher expression of PD-1 on peripheral CD8^+^ T cells detected in all the patients treated with TKI, especially for TKI-F patients, this consistent with recent research that a higher percentage of PD-1 detected on CD4^+^, CD8^+^ and Treg cells in CML patients resistant to TKI (33), however, on the level of T cell subsets, we can see that the percentage of PD-1 high expression T cell subsets mainly decreased in the patients who achieved MMR but not in TKI-F and Pre-MMR patients. Those results indicated that dynamic monitoring of the changes of these immune phenotypes in the level of T cell subsets may help to predict the effects and outcomes after TKI treatment.

PD-1 and TIGIT are two classic IC receptors that negatively modulate T-cell responsiveness and limit T-cell activation during antigen exposure (34–36). Consistent with previous studies, our results also demonstrated that the level of PD-1^+^CD8^+^ T cells increased in the PB but not BM of DN-CML patients (18, 37), but taking a close look at the subsets, we found that CD8^+^ T_EM_ and T_EMRA_ subsets were mainly impaired, while the CD4^+^ T_EM_ also affected. In the BM T cell subsets, the higher TIGIT expression was only found in the CD8^+^ T_EMRA_ subset in the TKI-F group but not in the total CD8^+^ level. T_EM_ and T_EMRA_ are the main effector subsets contributing to quickly clearing pathogens. The increased expression of PD-1 and TIGIT on these two subsets may attenuate their anti-leukemia function. A clinical trial aiming to improve the MMR ratio for TKI-F patients by adding anti-PD-1 nivolumab/pembrolizumab to TKI inhibitors has been completed, however, approximately 40% of the patients still were TKI treatment failed (NCT#02011945). Our data may help to discover more precise anti-leukemia immune therapy by focusing on studying the pathologic mechanism of the dysfunction of T_EM_ and T_EMRA_ subsets in the future.

Except for the classical memory T cell subsets, T_RM_ is a specific memory T cell located in unique tissue and organs, which provides a lifelong immune protective effect to the regional tissue (28, 38, 39). Increasingly studies have found that the quantity and quality of T_RM_ cells are critical targets for immunotherapeutic modulation and prognostic outcomes in tumor (14). However, there are still no reports that describe CD8^+^ T_RM_ alterations in the BM of CML patients. Here, we first time found that the number of CD8^+^ T_RM_ cells is significantly increased in the BM of DN-CML patients accompanied by a higher expression of TIGIT and PD-1, however, patients who received TKI treatment not shown the same pattern. This result indicates that BM T_RM_ cells from DN-CML patients may be impaired by the leukemia cell. Further study of the function of CD8^+^ T_RM_ cells from the BM of DN-CML patients and looking for the BM microenvironment mechanisms which lead to this result may help to understand more immune dysfunction mechanisms during the development of CML.

For the proper functioning of T cells, the co-stimulatory signal provided by DCs is an essential determinant. Through interaction with CD80 and CD86 on the DC surface, CD28 modulates T cell proliferation, differentiation, survival, and cytokine secretion (40, 41). Indeed, previous studies have found that CD80 may prefer to combine with PD-L1 and CTLA-4 if CD80 had an advantage in expression (42). Several studies have found that CTLA4 expression regulatory T cells accumulated in the leukemia environment of DN-CML patients, while CML cells increased the expression of PD-L1 (18, 33, 43). Therefore, though the expression of CD28^+^ T cells remains at a normal level, the decreased level of CD86^+^ DCs and the unbalanced ratio of CD80/CD86 may also prevent the activation of T cells in DN-CML patients. Further explore the mechanism of the downregulation of CD86 on DCs cells using RNA sequencing and other methods is necessary for developing DCs related immune treatment strategies.

**Figure 5:**
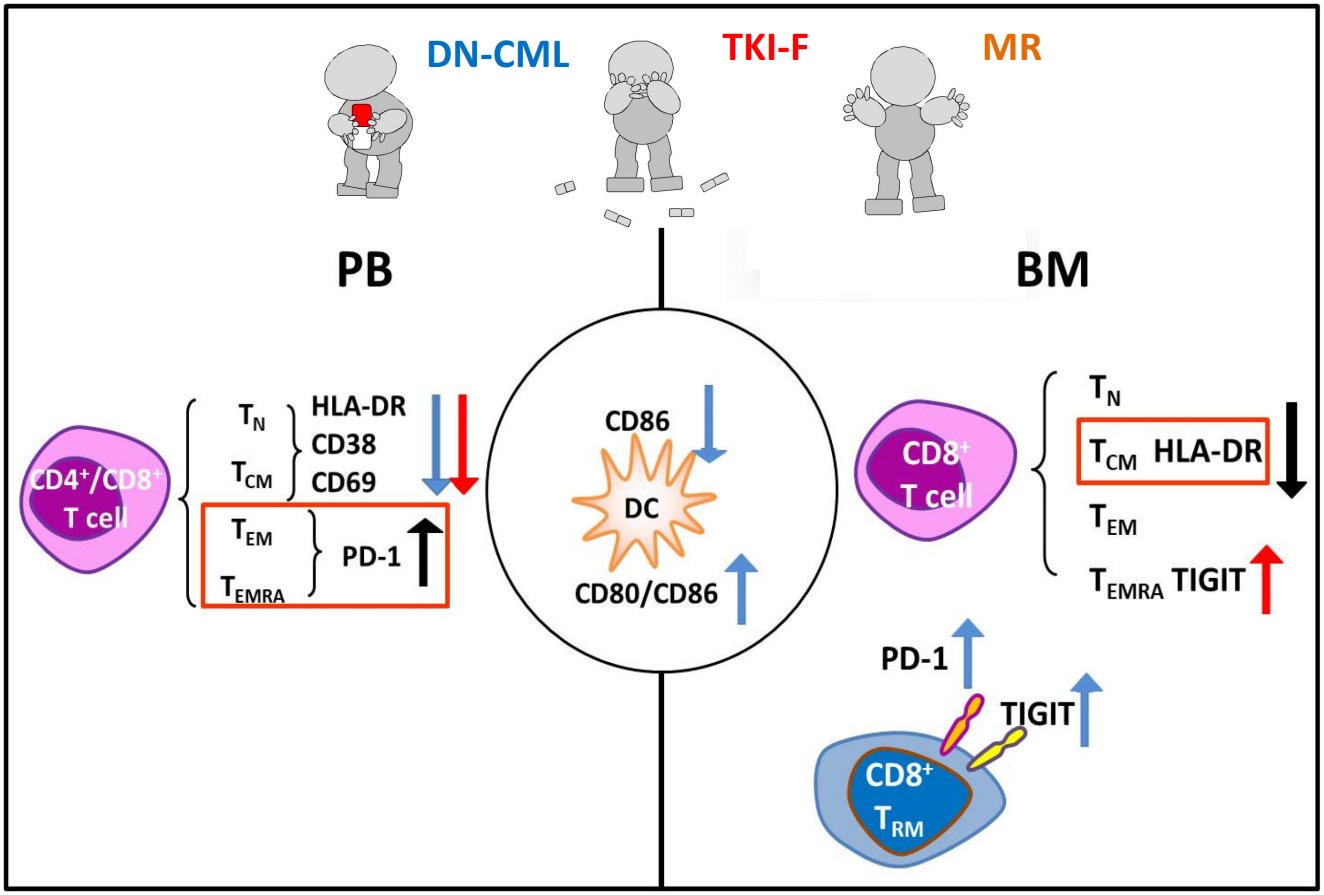
Model illustrating the immunophenotypic differences of the T cells subsets in PB and BM in HIs and CML groups. The left and right of the figure respectively show the alterations of PB and BM, and the center represents the common change in PB and BM. Blue, red, and black respectively represent alterations in DN-CML, TKI-F, and MR.

Here, we further observed that the early differentiated T cell subsets (T_N_, T_CM_) were inadequate activation and effector T cell subsets (T_EM_, T_EMRA_, T_RM_) exhibited diverse exhausted phenotypes in the PB and BM of CML patients with different disease statuses, which may impair T cells’ long-term immunological surveillance and simultaneously attenuate their ability to remove leukemia cells. Meanwhile, the DCs cells may be unable to valid stimulate T activation due to the decreased expression of CD86 and unbalanced CD80/CD86 ratio in DN-CML patients (**Figure 5**). These complex immune defects are worth further immune therapy strategy development. For example, immunotherapeutic methods not only need to inhibit PD-1 expression on effector T cells but also need to enhance the activation T_N_ and T_CM_ cells, as well as increase the co-stimulate function of DCs cells synergistically.

## Data availability statement

The original contributions presented in the study are included in the article further inquiries can be directed to the corresponding authors.

## Ethics statement

The studies involving human participants were reviewed and approved by Ethics Committee of the Medical School of Jinan University. The patients/participants provided their written informed consent to participate in this study. Written informed consent was obtained from the individual(s) for the publication of any potentially identifiable images or data included in this article.

## Author contributions

LX and YQL contributed to the concept development and study design. DY, LX, LL, and XBZ performed the laboratory studies. YHL, JZ, XFZ, XH, JW and XD collected the clinical information of patients. DY and LX drafted the manuscript. SC managed the laboratory reagents and financial affairs. DY, LX and YQL helped modify the manuscript. All authors contributed to the article and approved the submitted version.

## Supplementary material

Supplementary Figure 1 CD4^+^ or CD8^+^ T_N_ and T_CM_ cells from the PB of DN-CML patients have a lower percentage of the activation markers CD38 and HLA-DR, and this is further decreased in TKI-F patients. The frequency of CD38, CD69, and HLA-DR on CD4^+^ T_N_ **(A)**, CD4^+^ T_CM_ **(B)**, CD8^+^ T_N_ **(C)** and CD8^+^ T_CM_ **(D)** in the PB of HIs (PB, n = 12, BM, n = 8) and DN-CML (PB, n = 12, BM, n = 8), TKI-F (PB, n = 9, BM, n = 6) pre-MMR (CD4, n = 9, CD8, n = 9), and MMR (CD4, n = 8, CD8, n = 8) patients. The *P* values shown are from the Mann-Whitney U test between groups. Significance is indicated as *, *P* < 0.05 in red.

Supplementary Figure 2 A decreased level of HLA-DR^+^CD8^+^ T_CM_ cells exists in all CML groups, and TKI-F patients have increased expression of the exhaustion marker TIGIT on BM CD8^+^ T_EMRA_ cells. The frequency of CD38, CD69, HLA-DR, CD57, CD28, BTLA, TIGIT, and PD-1 in CD8^+^ T cells **(A)**, CD8^+^T_N_ **(B)**, CD8^+^T_CM_ **(C)**, CD8^+^T_EM_ and CD8^+^T_EMRA_ among HIs (n = 8) and DN-CML (n = 11), TKI-F (n = 6) and MR (n = 6) patients. The *P* values shown are derived from the Mann-Whitney U test between groups.
