# Supplementary figures and images for: Comprehensive analysis of the immune pattern of T cell subsets in chronic myeloid leukemia before and after TKI treatment
Source: Front Immunol. 2023 Jan 19;14:1078118. doi: 10.3389/fimmu.2023.1078118 (PMC9893006; doi:10.3389/fimmu.2023.1078118)

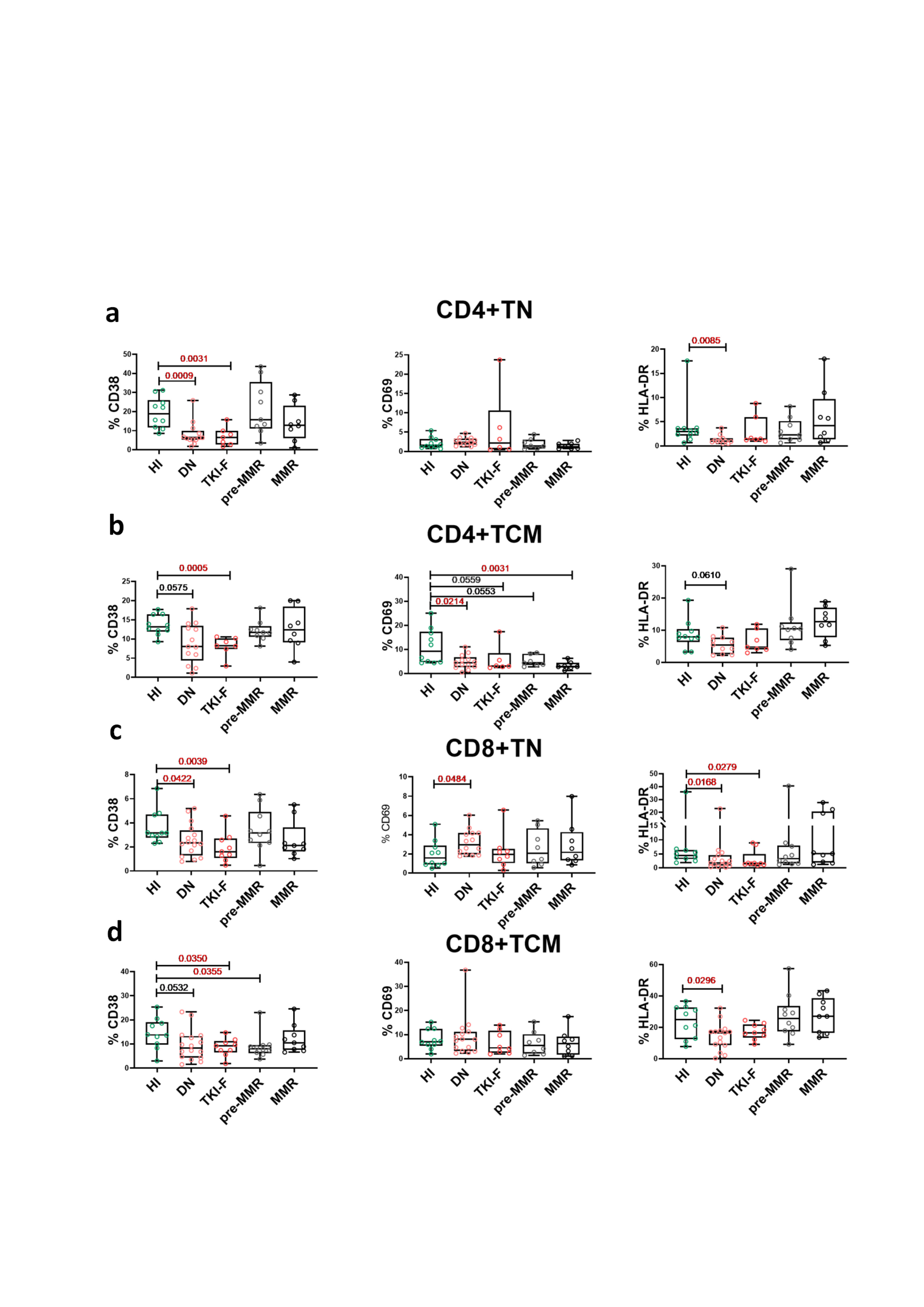

Supplement: Supplementary Figure 1 — CD4+ or CD8+ TN and TCM cells from the PB of DN-CML patients have a lower percentage of the activation markers CD38 and HLA-DR, and this is further decreased in TKI-F patients. The frequency of CD38, CD69, and HLA-DR on CD4+ TN (A), CD4+ TCM (B), CD8+ TN (C) and CD8+ TCM (D) in the PB of HIs (PB, n = 12, BM, n = 8) and DN-CML (PB, n = 12, BM, n = 8), TKI-F (PB, n = 9, BM, n = 6) pre-MMR (CD4, n = 9, CD8, n = 9), and MMR (CD4, n = 8, CD8, n = 8) patients. The P values shown are from the Mann-Whitney U test between groups. Significance is indicated as *, P < 0.05 in red. [file Image_1.jpeg]

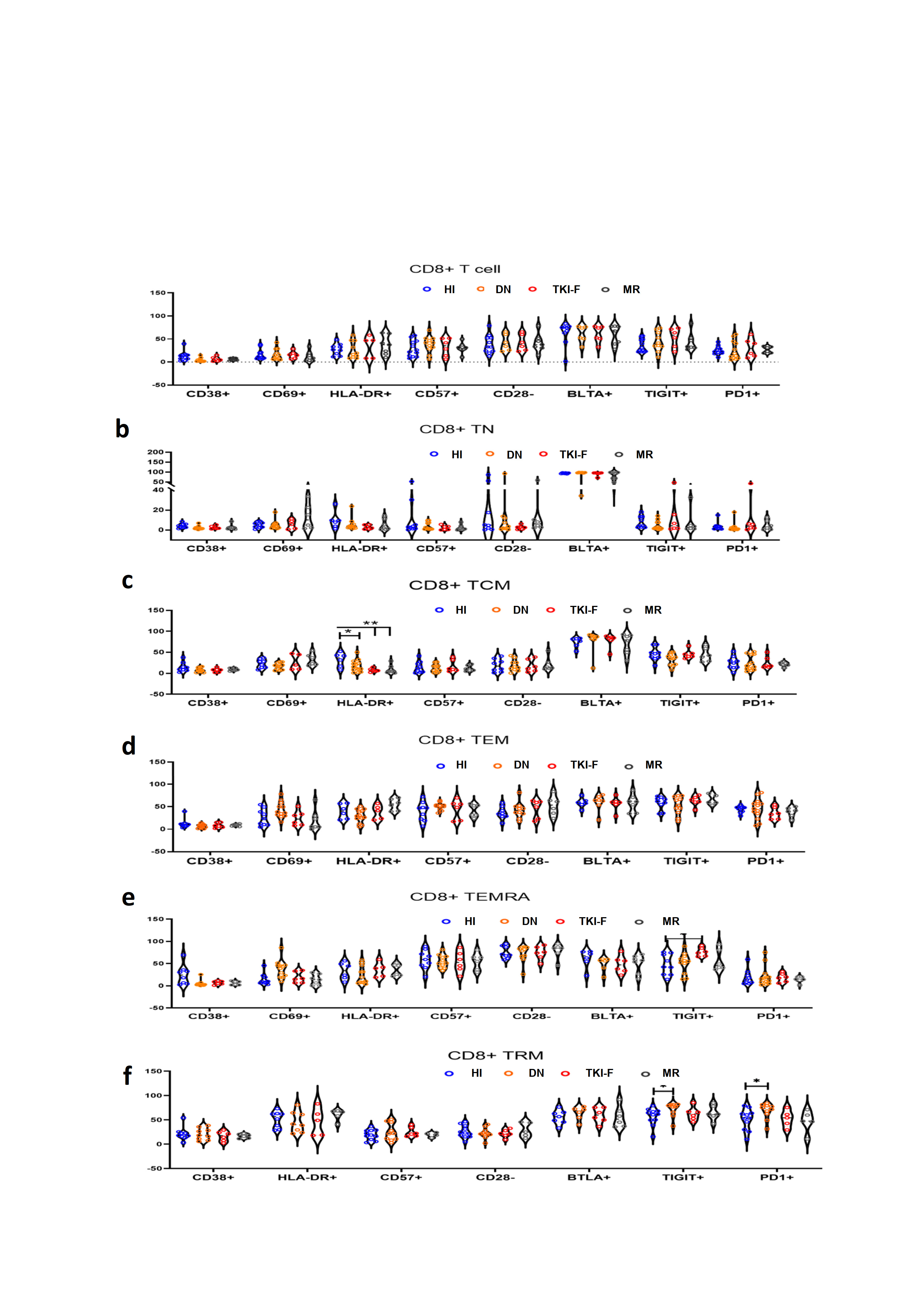

Supplement: Supplementary Figure 2 — A decreased level of HLA-DR+CD8+ TCM cells exists in all CML groups, and TKI-F patients have increased expression of the exhaustion marker TIGIT on BM CD8+ TEMRA cells. The frequency of CD38, CD69, HLA-DR, CD57, CD28, BTLA, TIGIT, and PD-1 in CD8+ T cells (A), CD8+TN (B), CD8+TCM (C), CD8+TEM and CD8+TEMRA among HIs (n = 8) and DN-CML (n = 11), TKI-F (n = 6) and MR (n = 6) patients. The P values shown are derived from the Mann-Whitney U test between groups. [file Image_2.jpeg]
