# Supplementary material for: Comprehensive analysis of the immune pattern of T cell subsets in chronic myeloid leukemia before and after TKI treatment
Source: Front Immunol. 2023 Jan 19;14:1078118. doi: 10.3389/fimmu.2023.1078118 (PMC9893006; doi:10.3389/fimmu.2023.1078118)
Supplement: Supplementary file 3 [file DataSheet_1.pdf]

Supplemental Table 1. MR PB sample characteristics

	Age/ Gender	Status	BCR-ABL1 (IS) %	TKI-duration （months）	TKI-drug
P1	38/F	MMR	No detected	45	Imatinib
P2	25/F	MMR	0.090	5	Imatinib
P3	21/F	MMR	0.090	9	Imatinib
P4	25/M	MMR	0.030	15	Nilotinib
P5	26/F	MMR	0.032	96	Dasatinib
P6	43/F	MMR	0.028	23	Imatinib
P7	79/M	MMR	0.020	24	Imatinib
P8	46/F	MMR	0.050	48	Imatinib
P9	47/M	MMR	0.050	108	Imatinib
P10	61/F	MMR	0.030	55	Imatinib
P11	60/F	Pre-MMR	5.960	83	Nilotinib
P12	79/M	Pre-MMR	0.194	5	Imatinib
P13	56/F	Pre-MMR	0.530	4	Dasatinib
P14	35/M	Pre-MMR	4.173	15	Dasatinib
P15	35/M	Pre-MMR	3.130	36	Imatinib
P16	39/M	Pre-MMR	0.210	24	Dasatinib
P17	33/M	Pre-MMR	5.960	3	Imatinib
P18	46/M	Pre-MMR	8.290	1	Imatinib
P19	28/M	Pre-MMR	0.112	6	Imatinib
P20	40/F	Pre-MMR	0.120	7	Imatinib

MMR = major molecular remission, Pre-MMR = the period before major molecular remission, IS = international standard
